# Exploring Social Media Use and Anxiety Symptoms in Health Care Workers During the COVID-19 Pandemic: Cross-Sectional Secondary Data Analysis

**DOI:** 10.2196/102008

**Published:** 2026-10-15

**Authors:** Melissa Powell, Kristen Choi, Russell G Buhr, Lauren E Wisk

**Affiliations:** 1 Division of General Internal Medicine and Human Services Research, The National Clinician Scholars Program, University of California, 1100 Glendon Ave Suite 900, Los Angeles, CA, 90024, United States, 310-853-0918; 2 Fielding School of Public Health, Department of Health Policy and Management, Joe C. Wen School of Nursing, University of California, Los Angeles, CA, United States; 3 Fielding School of Public Health, Health Policy and Management, Department of Medicine, University of California, Los Angeles, CA, United States; 4 Department of General Internal Medicine & Health Services Research, University of California, Los Angeles, CA, United States

**Keywords:** health care workers, anxiety, social media, digital behavior, COVID-19, mental health, factor analysis, occupational health

## Abstract

**Background:**

Health care workers (HCWs) experienced significant mental health challenges, particularly anxiety, during the COVID-19 pandemic. Although social media became a primary source of information and connection, it was also a potential source of stress. The influence of social media on HCWs’ anxiety is not well understood.

**Objective:**

This study examined associations between social media use and anxiety symptoms among HCWs during the COVID-19 pandemic.

**Methods:**

We conducted a secondary, cross-sectional analysis of data from the 2021 UC-COVID study (N=427 HCWs). Anxiety symptoms were assessed using the Generalized Anxiety Disorder-2 (GAD-2). Social media use across multiple platforms (Twitter, Facebook, Instagram, other social media platforms, and other media sources) was evaluated using factor analysis to create a single variable for social media use. Logistic regression models estimated associations between overall social media use and anxiety symptoms, both unadjusted and adjusted for demographic, occupational, and health-related characteristics.

**Results:**

Factor analysis supported a single latent factor representing overall social media use, with all platform indicators loading significantly on the factor (*P*<.001) and with moderate loadings (0.33‐0.59). Of 427 HCWs, the mean age was 44.3 (SD 13.5), and the majority identified as female (n=314, 73.5%) and as working in a hospital location (n=136, 43.6%). Facebook was used the most, with 54% (n=230) of participants using the platform at least once per day. A total of 29% reported clinically relevant anxiety symptoms (GAD-2 ≥3). Using the social media variable from the factor analysis, higher social media use was significantly associated with anxiety symptoms (odds ratio [OR] 1.77, CI 1.15‐2.73). Older age was significantly associated with reduced anxiety symptoms (adjusted odds ratio [aOR] 0.97, CI 0.95‐0.99). HCWs with a history of mental health diagnoses reported higher levels of anxiety symptoms (aOR 2.33, CI 1.35‐4.03). Non-Hispanic Asian HCWs reported fewer anxiety symptoms compared to Non-Hispanic White HCWs (aOR 0.39, CI 0.19‐0.80). Participants reporting higher income had significantly lower odds of anxiety symptoms than those in the lower-income group (aOR 0.51, CI 0.30‐0.87).

**Conclusions:**

Study findings highlight the multifactorial nature of anxiety symptoms in HCWs and the need to consider social media use as an important contributor. Integrating HCWs and multiplatform social media use into a single measure offered an innovative lens on digital behavior and anxiety symptoms. The rapidly evolving digital landscape underscores the need for continued research. Future studies should include emerging social media platforms (ie, TikTok, Reddit, YouTube) as usage patterns shift over time. Longitudinal and mixed methods approaches are necessary to understand patterns and methods of social media use over time. Larger and more diverse HCW samples, stronger mental health measures (eg, GAD-7, depression, and burnout scales), and analyses stratified by clinical role and sociodemographic factors will be essential to guide interventions supporting mental health and well-being.

## Introduction

### Background

Health care workers (HCWs) work stressful jobs and have been exposed to higher levels of burnout in the aftermath of the COVID-19 pandemic [1]. Nearly half of HCWs reported experiencing burnout as of 2022, compared to one-third in 2018, and turnover intention among HCWs increased by 11% from 2018 to 2022 [1]. The COVID-19 pandemic led to increased isolation and negatively affected mental and physical health [2], both of which are important for protecting against burnout and maintaining general well-being [2]. One understudied factor regarding HCWs’ well-being in the COVID-19 pandemic has been the impact of social media use. Examining the relationships between social media use and anxiety symptoms among HCWs may shed new light on potential contributing factors affecting mental health in the current technological landscape.

### Review of Relevant Scholarship

Internet addiction, which includes 24/7 access to and frequent use of media, including social media use, has been shown to contribute to mental health conditions [3]. During the COVID-19 pandemic, the media became less positive and included discussions of loneliness, anxiety, and depression across media sources [1,2,4]. Incidents of violence and aggression toward HCWs increased during the COVID-19 pandemic [5,6], with patients being influenced by content found online [6]. Additionally, information overload stemming from the media more broadly has been connected to COVID-19 fatalism and COVID-19 exhaustion [7]. In contrast, data exist that highlight potential benefits of social media use for HCWs by providing outlets for education, connection, and health promotion, but finding the right balance of exposure is crucial [8] and not yet defined. Managing the volume of social media content available, or the time spent consuming content, is particularly hard to measure [8].

Knowledge of social media’s impact on HCWs is generally limited, specifically in the context of the COVID-19 pandemic, although one study found high levels of anxiety and fear (over 50% of their sample), with social media use being the most influential variable on anxiety [9]. The extent of social media use and the specific types of use are not frequently surveyed; media are often studied broadly, including news and print media and are less frequently studied in HCW samples. Mainstream media, including social media and news outlets, have been analyzed to look at factors like diversity and representation among HCWs that are featured across media sources [10], but this work has not focused on the role of HCW media consumption in mental health outcomes. Although some HCW groups, such as emergency medicine physicians [4], have been studied to understand the impact of media, many HCW groups have not been researched nor compared to one another to understand any differences in the experience of anxiety. A literature review of nurses and social media use related to the COVID-19 pandemic found that mostly cross-sectional surveys were conducted; social media use was not often the main variable of interest, the populations varied widely, and mental health outcomes were not measured [11].

Sociodemographic factors have been identified as important correlates of anxiety in studies examining mental health during the COVID-19 pandemic, including among HCWs. One study suggested that females reported greater fear of COVID-19 infection [9]. Another study found that adults younger than 35 years were more likely to experience anxiety and depression compared with older adults [12]. Additionally, a systematic review found that frequent media consumption related to the COVID-19 pandemic was associated with increased anxiety, while gender, age, and the presence of chronic illness were also associated with poorer mental health outcomes [13]. Together, these findings suggest that sociodemographic characteristics may contribute to differences in mental health outcomes and should be considered when developing interventions to support HCWs.

More recent evidence since the onset of the COVID-19 pandemic suggests that HCWs continue to experience elevated levels of anxiety, psychological distress, and emotional exhaustion beyond the acute phase of the COVID-19 pandemic [14-16]. At the same time, HCWs increasingly engage with social media and digital platforms for information, professional communication, and social connection, yet the influence of social media use on mental health outcomes remains poorly understood [17]. Existing research has often focused on specific professional groups or treated broad media use as a secondary variable, leaving limited understanding of how social media use is associated with anxiety across diverse groups of HCW disciplines [16,17].

### Study Aims

The study aims of this secondary analysis were broadly framed around exploring the following research question: what associations exist between social media use and anxiety levels among HCWs during the COVID-19 pandemic while controlling for sociodemographic factors?

## Methods

### Design

We conducted a secondary, cross-sectional analysis of data from the first time point of the University of California, Los Angeles’ (UCLA’s) UC-COVID (understanding community considerations, opinions, values, impacts, and decisions for COVID-19) Study [18,19]. The UC-COVID Study [18,19] is a community engagement study that included an internet-based survey conducted at three time points from 2020 to 2022; data for this analysis are from the baseline survey, which was conducted during the acute period of the COVID-19 pandemic in 2020.

### Inclusion and Exclusion Criteria

Participants for the baseline survey were adults aged 18 years or older. For our secondary analysis, participants were included if they identified as an HCW (n=582). Participants were excluded from the analysis if complete data were not available for the primary exposure and outcome variables (n=32 were excluded). Participants with missing data for only the primary exposure (n=94 for missing data and n=21 for “prefer not to answer” for social media use) or only the outcome variables (n=3 for missing data and n=5 for “prefer not to answer” for anxiety symptoms) were also excluded, resulting in a final analytic sample of 427. Noted, the majority of missing data resulted from survey attrition prior to reaching the section of the survey that included data on social media use, which was toward the end of the survey.

### Participant Characteristics

Survey participants self-reported demographic data (eg, age, gender, race, ethnicity, income, religion, and politics), work roles, health status and chronic conditions, social and behavioral habits, media use, and values and beliefs related to the COVID-19 pandemic.

### Sampling Procedure

For the UC-COVID Study, participants were originally recruited from May to September 2020 through social media platforms (eg, Facebook, Twitter) and direct recruitment through organizations (eg, University of California, University of California Health Systems, and AltaMed) and professional societies (eg, Society for General Internal Medicine).

### Sample Size, Power, and Precision

The sample size for this study (N=427) was determined by the availability of participants in the existing dataset for this secondary analysis; no a *priori* power analysis was conducted to determine sample size. To contextualize the adequacy of the sample, a post hoc power analysis was conducted using G*Power 3.1 (Heinrich Heine University Düsseldorf) to determine the smallest effect size detectable given the achieved sample size, assuming a logistic regression framework. With *α*=.05, 80% power, an outcome prevalence of 29%, and an *R*² of 0.12 between the social media use predictor and other model covariates, the study had adequate power to detect an odds ratio (OR) of 1.38 (or its inverse, 0.72) for the association between social media use and anxiety. Effects smaller than this threshold would not have been reliably detected at conventional power levels given the available sample.

### Measures and Covariates

To assess our primary outcome, anxiety symptoms, we used the 2-item version of the Generalized Anxiety Disorder screening tool (GAD-2). This tool assesses the frequency of anxiety symptoms in the past 2 weeks: (1) feeling nervous, anxious, or on edge, and (2) being unable to stop or control worrying. These items are rated on a 0-3 scale from not at all to nearly every day. Items are summed; a score of 3 or greater is clinically suggestive of anxiety [20].

To measure social media use (the primary exposure), participants reported how often they used various online platforms to obtain news or information. Five items assessed the frequency of engagement with the following: (1) Twitter, (2) Instagram, (3) Facebook, and (4) other social media platforms (for which participants could specify the platform), and (5) other online video websites (eg, YouTube and TikTok). Each item used the same response scale, ranging from: Several times a day, About once a day, A few times a week, Every few weeks, Less often than every few weeks, to I never use this platform. Responses were coded such that higher values indicated greater use of the platform.

We assessed several sociodemographic characteristics to examine additional correlates of anxiety symptoms. HCW type was categorized into 4 groups: physicians and advanced practice providers, registered nurses and medical assistants, clinical support services (ie, respiratory therapists, physical therapists, or social workers), and other HCWs which included administrative HCWs and workers in adjacent health care settings (ie, veterinarians or dentists). Workplace setting (HCW location) was classified as hospital-based, outpatient, or other/not currently working (ie, other settings such as schools or public health). Age was treated as a continuous variable. Sex was coded as a binary measure: male and female. Race and ethnicity were captured using a combined four-level variable due to small group sizes: (1) non-Hispanic/White, (2) non-Hispanic/non-White (including Black/African American, American Indian/Alaska Native, and multiracial), (3) non-Hispanic/Asian (including Asian and Native Hawaiian/Pacific Islander), and (4) Hispanic/Latino. Educational attainment was dichotomized into less than a bachelor’s degree vs a bachelor’s degree or higher. Annual household income was categorized into 2 income brackets: less than US $100,000 annually, or US $100,000 or greater annually, which was approximately the median household income level in the state of California [21]. Political affiliation was operationalized as 4 categories: conservative, moderate, liberal, and nonpolitical. Religious affiliation was captured through an indicator reflecting endorsement of any religious identification vs none (including agnostic and atheist). Finally, participants reported whether they had a history of a mental health diagnosis, coded as a binary variable.

### Missing Data

Participants with missing data for the primary exposure and outcome variables were excluded (n=32); participants with missing data for only the primary exposure (n=94 for missing data and n=21 for “prefer not to answer” for social media use) or only the outcome variables (n=3 for missing data and n=5 for “prefer not to answer” for anxiety symptoms) were also excluded. For all other covariates with missing data (ie, work location, political affiliation, religious affiliation, and income), a missing data category was used to retain the analytic sample size of 427. The use of missing categories increases the potential for residual confounding due to misclassification of covariates with missing data–a limitation of this study. Additionally, to characterize the missing-data mechanism for the primary exposure and outcome, we conducted Little’s missing completely at random test [22], separately for the exposure and core covariates and for the outcome, and examined whether missingness tracked the survey platform’s section-completion indicators (Multimedia Appendix 1).

### Analytic Strategy

Statistical analyses were conducted in Stata (StataCorp, LLC; version 18.0). Our significance level was set at a two-sided α of .05. A confirmatory factor analysis (CFA) was conducted to evaluate whether 5 indicators of social media use—Twitter, Instagram, Facebook, other social media, and other internet use—reflected a unified latent construct of social media use. All indicators were specified as reflective measures loading onto a single latent factor (based on the results of an exploratory factor analysis) for which higher values indicated greater social media use, and standardized coefficients were estimated to assess model fit.

Descriptive statistics and appropriate bivariate tests (continuous variables were assessed using 2-tailed *t* tests, and categorical variables were compared using chi-square tests or Fisher exact tests, as appropriate given group sizes) were used to describe the prevalence of symptoms of anxiety by various characteristics The frequency of social media use by platform was descriptively reported in Table 1. We estimated a series of logistic regression models to estimate the association between social media use and anxiety, both unadjusted and adjusted for covariates (Table 2). The social media use score derived from the CFA was treated as a continuous variable; ORs represented the change in odds of anxiety symptoms per one-unit (approximately one SD) increase in overall social media use. To construct a parsimonious adjusted model, covariates found to be significantly associated with anxiety symptoms in bivariate analyses were included.

**Table 1.** Frequency of social media (SM) use by platform (N=427)^a^.

Platform	Several times a day, n (%)	Once a day, n (%)	A few times a week, n (%)	Every few weeks, n (%)	Less often than every few weeks, n (%)	Never, n (%)
Twitter (X)	50 (11.7)	36 (8.4)	32 (7.5)	33 (7.7)	42 (9.8)	234 (54.8)
Instagram	92 (21.6)	57 (13.4)	47 (11.0)	28 (6.6)	51 (11.9)	152 (35.6)
Facebook	139 (32.6)	91 (21.3)	55 (12.9)	26 (6.1)	35 (8.2)	81 (19.0)
Other SM	26 (6.1)	38 (8.9)	49 (11.5)	26 (6.1)	30 (7.0)	258 (60.4)
Other internet	33 (7.7)	42 (9.8)	78 (18.3)	45 (10.5)	58 (13.6)	171 (40.1)
SM use score (mean SD; min-max)	—^b^	—	—	—	—	0.0 ( 0.48; –0.78 to 1.28 )

a The frequency of social media use across the platforms, with usage frequencies of several times a day, once a day, a few times a week, every few weeks, less often than every few weeks, or never. The SM use score is the single latent variable derived from the factor analysis.

b Not applicable.

**Table 2.** Associations between social media use and anxiety symptoms.^a^

Variable	Model 1		Model 2		
	OR^b^ (95% CI)	*P* value	OR (95% CI)		*P* value
Social media use	1.77 (1.15-2.73)	.009	1.74 (1.08‐2.83)		.02
Age	—^c^	—	0.97 (0.95‐0.99)		.01
Race/ethnicity (reference: White)					
Non-Hispanic non-White	—	—	0.77 (0.29‐2.02)		.60
Non-Hispanic Asian	—	—	0.39 (0.19‐0.80)		.01
Hispanic/Latino	—	—	1.25 (0.66‐2.41)		.36
HCW^d^ location (reference: hospital)					
Outpatient	—	—	1.55 (0.89‐2.71)		.12
Other/not working	—	—	1.05 (0.56‐1.98)		.88
Missing	—	—	0.46 (0.08‐2.82)		.40
Chronic MH^e^ condition (reference: no MH)					
Yes, MH condition	—	—	2.33 (1.35‐4.03)		.002
Political affiliation (reference: conservative)					
Moderate	—	—	1.20 (0.54‐2.67)		.66
Liberal	—	—	0.58 (0.29‐1.14)		.11
Nonpolitical	—	—	0.70 (0.23‐2.17)		.54
Missing	—	—	1.41 (0.29‐6.96)		.67
Income level (reference: low income)					
High income	—	—	0.51 (0.30‐0.87)		.01
Missing	—	—	0.34 (0.10‐1.17)		.09

a The logistic regression results showing associations between social media use (using our single latent variable from factor analysis) and anxiety symptoms. Model 1 includes only the exposure and outcome variables in the model, while model 2 includes sociodemographic variables. The reference group for each variable has been added in parentheses next to the variable name. The significance level for the *P* value was set α to .05.

b OR: odds ratio.

c Not applicable.

d HCW: health care worker.

e MH: mental health.

### Ethical Considerations

This study used data from the UC-COVID Study, which was approved by the UCLA Institutional Review Board (IRB #20‐000683) and registered at ClinicalTrials.gov (NCT04373135). All participants in the original study provided informed consent via an online consent form prior to completing the baseline survey; this consent and the governing IRB protocol permitted secondary data analysis with deidentified data to protect participant confidentiality. Participants in the original study who provided an email address were entered into a gift card raffle, with entries awarded based on the completion of follow-up surveys. No additional compensation was provided for this secondary analysis. This manuscript and any supplementary materials do not include any images or figures depicting participants or identifiable individuals.

## Results

### Missing Data Analysis

Both missing completely at random tests [22] rejected the null hypothesis of missing completely at random (exposure/covariates: *χ*_5_²=22.5; *P*<.001; outcome: *χ*_12_²=47.5; *P*<.001); the large majority of missingness on these items (n=125, 98.4% for social media use) was attributable to survey noncompletion rather than item-level nonresponse, consistent with missing at random conditional on completion status (Multimedia Appendix 1).

### Frequency of Social Media Use by Platform

Table 1 shows the frequency of social media use across platforms. Twitter use was generally low, with more than half of participants reporting that they never used it for news (234/427, 54.8%). Instagram use was more evenly distributed, though over one-third reported never using it (152/427, 35.6%). Facebook had the highest levels of frequent engagement, with 32.6% (139/427) using it several times per day and another 21.3% (91/427) using it about once per day. In contrast, most participants reported never using other social media platforms (258/427, 60.4%). Use of other internet sites (eg, YouTube or TikTok) was more diffuse, with participants reporting use only a few times per week (78/427, 18.3%) or never (171/427, 40.1%). Participants most frequently listed LinkedIn, Reddit, Snapchat, TikTok, and YouTube as the other platforms that they commonly used (based on free-text responses).

### Factor Analysis Results

Table 3 shows the results of the factor analysis. All 5 social media use items demonstrated strong and statistically significant standardized factor loadings on the latent factor, with moderate coefficients ranging from 0.33 to 0.59. The highest loading was observed for other internet use (standardized loading=0.59; *P*<.001). Model fit was acceptable (*χ*²_5_=11.2; *P*=.047; Comparative Fit Index (CFI) 0.96, Tucker-Lewis Index (TLI) 0.91, root mean square error of approximation, RMSEA 0.05).

**Table 3.** Factor analysis of social media (SM) use variables^a^.

Variable	Factor loading (95% CI)	OIM^b^ SE	*z* score	*P* value
Twitter^c^	0.334 (0.216-0.451)	0.06	5.54	<.001
Instagram^d^	0.41 (0.302-0.535)	0.059	7.05	<.001
Facebook^e^	0.358 (0.242-0.474)	0.059	6.03	<.001
Other SM^f^	0.54 (0.427-0.653)	0.058	9.36	<.001
Other internet^g^	0.588 (0.474-0.702)	0.058	10.07	<.001

a The factor loadings and residual variances from a factor analysis of social media use variables. LR (Likelihood ratio) test of the model vs saturated: *χ*_5_²=11.2; *P*=.047. All factor loadings were statistically significant at *P* value <.001.

b OIM: Observed Information Matrix.

c Residual variance: 0.889 (95% CI 0.813-0.971).

d Residual variance: 0.825 (95% CI 0.733-0.928).

e Residual variance: 0.872 (95% CI 0.792-0.959).

f Residual variance: 0.709 (95% CI 0.597-0.842).

g Residual variance: 0.654 (95% CI 0.533-0.804).

### Sample Characteristics

Table 4 shows the sample characteristics. HCWs were predominantly either medical doctors (MDs), doctors of osteopathic medicine (DOs), or advanced practice providers (APPs) at 35.6% (n=152) or registered nurses (RNs) or medical assistants (MAs; RNs/MAs) at 23.2% (n=99; Table 4). Clinical support service personnel (n=132, 30.9%) included HCWs identifying as respiratory therapists, physical therapists, or social workers, for example. Other HCWs (n=44, 10.3%) included primarily administrative HCWs and workers in adjacent health care settings (eg, veterinarians or dentists). The average age of HCWs was 44.3 (SD 13.5) years and HCWs were primarily female (n=314, 73.5%), Non-Hispanic white (n=262, 61.4%), had a bachelor’s degree or more (n=390, 91.3%), household income of US $100,000 dollars and greater per year (n=246, 57.6%), liberal (n=239, 56.0%), and affiliated with a religion (n=270, 63.2%). Around 21.1% (n=90) of HCWs reported having a prior history of mental health condition diagnosis.

**Table 4.** Health care worker (HCW) characteristics and anxiety symptoms (N=427)^a^.

Variable	Significant anxiety symptoms (GAD-2^b^ ≥3)	Nonsignificant anxiety symptoms	Total	*P* value^c^
Total, n (%)	125 (29.3)	302 (70.7)	427 (100)	
Age (y), mean (SD)	40.3 (11.9)	46.0 (13.7)	44.3 (13.5)	<.001
Sex, n (%)				.01
Male	40 (35.4)	73 (64.6)	113 (26.5)	
Female	85 (27.1)	229 (72.9)	314 (73.5)	
HCW^d^ type, n (%)				.16
MDs^e^, DOs^f^, and APPs^g^	42 (27.6)	110 (72.4)	152 (35.6)	
RNs^h^/MAs^i^	23 (23.2)	76 (76.8)	99 (23.2)	
Clinical support services^j^	48 (36.4)	84 (63.6)	132 (30.9)	
Other HCWs^k^	12 (27.3)	32 (72.7)	44 (10.3)	
HCW location, n (%)				.02
Hospital	50 (26.9)	136 (73.1)	186 (43.6)	
Outpatient	46 (39.3)	71 (60.7)	117 (27.4)	
Other/not working	27 (23.9)	86 (76.1)	113 (26.5)	
Missing	2 (18.2)	9 (81.8)	11 (2.6)	
Race/ethnicity^l^, n (%)				.02
Non-Hispanic White	82 (31.3)	180 (68.7)	262 (61.4)	
Non-Hispanic non-White	7 (26.9)	19 (73.1)	26 (6.1)	
Non-Hispanic Asian	12 (15.6)	65 (84.4)	77 (18.0)	
Hispanic/Latino	24 (38.7)	38 (61.3)	62 (14.5)	
Education level, n (%)				.66
Less than a bachelor’s degree	12 (32.4)	25 (67.6)	37 (8.7)	
A bachelor’s degree or more	113 (29.0)	277 (71.0)	390 (91.3)	
Income level, n (%)				<.001
Annual household income less than 100,000	55 (45.8)	65 (54.2)	120 (28.1)	
Annual household income of 100,000 or greater	56 (22.8)	190 (77.2)	246 (57.6)	
Missing	14 (23.0)	47 (77.0)	61 (14.3)	
Political affiliation, n (%)				.004
Conservative	26 (44.8)	32 (55.2)	58 (13.6)	
Moderate	26 (38.2)	42 (61.8)	68 (15.9)	
Liberal	56 (23.4)	183 (76.6)	239 (56.0)	
Nonpolitical	7 (29.2)	17 (70.8)	24 (5.6)	
Missing	10 (26.3)	28 (73.7)	38 (8.9)	
Religious affiliation, n (%)				.10
Religious	87 (32.2)	183 (67.8)	270 (63.2)	
Nonreligious	28 (23.9)	89 (76.1)	117 (27.4)	
Missing	10 (25.0)	30 (75.0)	40 (9.4)	
Mental health diagnosis, n (%)				.01
Yes	36 (40.0)	54 (60.0)	90 (21.1)	
No	89 (26.4)	248 (73.6)	337 (78.9)	

a Participant characteristics across the entire sample and by their anxiety symptoms status based on GAD-2 scores (significant anxiety was reported if GAD-2 ≥3). Column percentages are shown in the “Total” column, while row percentages are shown elsewhere.

b GAD-2: Generalized Anxiety Disorder-2.

c Significance level for *P* values was set to α of .05.

d HCW: health care worker.

e MDs: medical doctors.

f DO: doctor of Osteopathic Medicine.

g APP: advanced practice provider.

h RN: registered nurse.

i MA: medical assistant.

j Clinical support services: respiratory therapists, physical therapists, and social workers, etc.

k Other HCWs: administrative workers and HCWs in adjacent health care settings (ie, veterinarians or dentists).

l Race and ethnicity were captured using a combined four‑level variable due to small group sizes: Non-Hispanic/White, Non-Hispanic/Non-White (including Black/African American, American Indian/Alaska Native, and multiracial), Non-Hispanic/Asian (including Asian and Native Hawaiian/Pacific Islander), and Hispanic/Latino.

### Regression Models

Table 2 shows the regression models. The unadjusted odds of reporting anxiety were significantly higher among those with higher *overall social media use* (OR 1.77, CI 1.15‐2.73; Table 2). In the adjusted model, high social media use remained associated with higher odds of anxiety (adjusted odds ratio [aOR] 1.74, CI 1.08‐2.83). Older age was significantly associated with lower odds of anxiety (aOR 0.97, CI 0.95‐0.99). Non-Hispanic Asian HCWs were significantly less likely to report anxiety symptoms compared to their Non-Hispanic white counterparts (aOR 0.39, CI 0.19‐0.80). Reporting a chronic mental health condition was also associated with elevated odds of reporting anxiety symptoms (aOR 2.33, CI 1.35‐4.03). Participants reporting a higher level of income had significantly lower odds of anxiety symptoms compared to the lower income group (aOR 0.51, CI 0.30‐0.87).

## Discussion

### Principal Findings

Our findings suggest that greater social media use among HCWs was associated with elevated anxiety symptoms during the COVID-19 pandemic. These results highlight the potential mental health implications of social media use among HCWs and underscore the importance of understanding how digital platforms may influence psychological well-being in health care settings.

These findings are consistent with prior research demonstrating associations between media use, increased information exposure, and anxiety, depression, and psychological distress in the general population during the pandemic [13,23,24]. Recent studies have also shown that HCWs continued to experience substantial mental health challenges, including anxiety, emotional distress, and burnout, beyond the acute phase of the pandemic [14-16]. At the same time, social media use plays an increasingly important role in information seeking, professional communication, and social connection among HCWs [17]. Although digital platforms provide opportunities for education, networking, and support, evidence suggests that their effects on mental health and well-being may be complex [25]. Our findings add to growing concerns regarding the potential mental health implications of social media use and extend the literature by linking social media use to anxiety symptoms among HCWs. Given the cross-sectional nature of this study and the limited assessment of the frequency of social media use, future research should use longitudinal designs and more detailed measures of social media use to better understand patterns of use, directionality, and relationships with mental health outcomes, potentially leveraging emerging AI and large language models to examine digital behaviors over time [26,27].

In our analysis, older age was a protective factor for lessened anxiety symptoms, independent of social media use. Current literature has shown that younger adults experienced higher levels of anxiety, depression, and psychological distress during the COVID-19 pandemic [13,28]. One reason for this finding is that older adults may possess more established coping strategies, greater emotional regulation, and increased resilience gained through life and professional experiences, which may help mitigate the psychological impact of stressful events [15]. Continued research exploring the connection between age and anxiety while using social media is warranted.

The significant association between race and ethnicity and anxiety symptoms among HCWs highlights the importance of considering potential cultural and structural factors that impact vulnerable populations and mental health outcomes. Non-Hispanic Asian participants had significantly lower odds of reporting anxiety than their White counterparts. This finding is consistent with national US surveillance data showing that Asian adults report lower levels of anxiety symptoms than other racial/ethnic groups [29]. Researchers have suggested that cultural differences in symptom expression, emotional restraint, stigma, and help-seeking behaviors may contribute to these observed differences [30,31]. Larger studies examining diverse racial and ethnic groups will help provide a deeper understanding of the relationship between minoritized groups and anxiety symptoms.

Finally, having a mental health diagnosis was significantly associated with anxiety symptoms among HCWs. This finding is consistent with prior research demonstrating that HCWs experienced elevated rates of anxiety, depression, and psychological distress during the COVID-19 pandemic and that individuals with existing mental health conditions may be particularly vulnerable to worsening symptoms during periods of prolonged stress [2,9,13,14,16,32]. One challenge with our finding is the nuance within the survey item. While the survey asked whether a previous mental health condition had been diagnosed, the ongoing medical management of the condition, including severity of the condition and medication use, was not assessed. These factors may influence the presence and severity of anxiety symptoms and could partially explain variation in outcomes. Future research should incorporate more comprehensive assessments of mental health history, treatment engagement, and other chronic health conditions to better understand how existing vulnerabilities influence psychological well-being among HCWs.

Higher income was associated with lower odds of anxiety symptoms when compared with the lower-income group, consistent with prior research demonstrating protective effects of financial stability on mental health [33,34]. Access to resources, health care, and buffers against stressors may be contributing factors when considering this association. Reporting of income commonly has high item nonresponse rates, which may have contributed to missing income data [35].

### Limitations

Limitations of this work include the cross-sectional nature of this analysis. One of the recruitment strategies for the UC-COVID Study included the use of social media platforms, which may have more readily recruited social media users. Although the survey asked participants about their frequency of social media use, it did not assess how participants engaged with social media sources (eg, what content is consumed), and further studies may be able to explore the details of how social media is used and the influence it may have on anxiety symptoms. Because missingness on our primary exposure and outcome was concentrated among participants who did not complete the survey, we cannot fully rule out that attrition itself relates to the underlying constructs of interest in a long, internet-administered survey. Our variables assessing social media use frequency should be interpreted cautiously and may not fully capture the underlying digital experiences contributing to anxiety symptoms, as prevalence of platform use may not represent actual user exposure that is shaped by personalized recommendations and engagement behaviors [36]. These data were also collected during the COVID-19 pandemic, which may have influenced responses during a stressful time globally, but it alternatively represents a unique time point, a stressful situation that may similarly occur again in the future (ie, pandemics, epidemics, and natural disasters impacting health care). Questions regarding income, religion, and politics, which individuals may not want to disclose, may have contributed to some residual confounding. As the sample is not population-based, generalizability is limited. Additionally, some HCW groupings (eg, RNs/MAs) could not be disaggregated, limiting inferences that can be made from heterogeneous groups. Although the most used social media platforms (Facebook, Twitter, and Instagram) were individually assessed at the time of surveying participants, additional social media platforms should be individually assessed due to the rapidly changing landscape and increase in new platforms, as evidenced by our highest factor loading scores for other forms of social media use. Another challenge that exists is the limited data available regarding anxiety symptoms that can be captured with the GAD-2, and the GAD-7 may provide more specific detailed data for analyzing anxiety outcomes [20,37].

### Conclusion

Findings from this study highlight the multifactorial nature of anxiety symptoms in HCWs and suggest that social media use may be an important factor associated with psychological well-being in this population. Higher levels of social media use were associated with increased anxiety symptoms, even after adjusting for several sociodemographic characteristics. By capturing social media use as a single composite exposure across a sample of HCWs, rather than focusing on one platform or professional group, as in previous research, this study offers new insight into how social media use relates to HCWs’ anxiety symptoms. Social media use should be considered when identifying ways to support HCWs’ mental health. Attention may be warranted for younger HCWs, those with lower incomes, and individuals with preexisting mental health conditions, who may be more vulnerable to experiencing anxiety symptoms independent of social media use. Future research should further explore the mechanisms influencing these relationships and consider longitudinal approaches to understand fluctuations in a larger, diverse sample of HCWs and the trajectory of social media use and its impact on mental health in a postpandemic health care landscape.

## Supplementary material

10.2196/102008 Multimedia Appendix 1 Missing data analysis tables.

## Abbreviations

aOR: adjusted odds ratio
APP: advanced practice provider
CFA: confirmatory factor analysis
CFI: Comparative Fit Index
DO: doctor of osteopathic medicine
GAD-2: Generalized Anxiety Disorder-2
HCW: Health care worker
IRB: Institutional Review Board
MD: medical doctor
medical assistant: medical assistant
OR: odds ratio
RMSEA: root mean square error of approximation
RN: registered nurse
TLI: Tucker-Lewis Index
UCLA: University of California, Los Angeles
