# Supplementary material for: Exploring Social Media Use and Anxiety Symptoms in Health Care Workers During the COVID-19 Pandemic: Cross-Sectional Secondary Data Analysis
Source: J Med Internet Res. 2026 Oct 15;28:e102008. doi: 10.2196/102008 (PMC13671597; doi:10.2196/102008)
Supplement: Supplementary file 1 [file jmir-v28-e102008-s001.docx]

**Missing Data Analysis Supplementary Tables**

Both MCAR tests rejected the null hypothesis of missing completely at random (exposure/covariates: χ²(5)=22.54, p=0.0004; outcome: χ²(12)=47.49, p<0.0001); the large majority of missingness on these items (98.4% for social media use) was attributable to survey non-completion rather than item-level nonresponse, consistent with missing at random conditional on completion status.

# **Table S1.** Missingness by Survey Section

Percent missing for each variable, grouped by the survey section in which it was administered (N = 582). Section 6 is the final section of the baseline survey.

| **Variable** | **Survey Section** | **N Missing** | **% Missing** |
| --- | --- | --- | --- |
| age | 1 | 0 | 0% |
| race_whit | 1 | 16 | 2.7% |
| chronic_12 | 2 | 0 | 0.0% |
| gad_q1 | 2 | 37 | 6.4% |
| gad_q2 | 2 | 41 | 7.0% |
| social_1 | 6 | 127 | 21.8% |
| politics | 6 | 171 | 29.4% |
| income | 6 | 194 | 33.3% |

# **Table S2.** Decomposition of Item Missingness by Own-Section Completion Status

For each variable, missingness is broken out by the completion status of the survey section in which it was administered (age, race_whit: Section 1; gad_q1, gad_q2, chronic_12: Section 2; social_1, politics, income: Section 6). comp=0: no responses in section (did not reach it); comp=1: partial completion; comp=2: fully completed; comp=3: a distinct routing category (Section 6 only). Cells marked n/a indicate that completion category did not occur for that section. The final column shows the percent of each variable's total missingness attributable to respondents who never reached that section, as opposed to genuine item-level nonresponse among those who completed it.

| **Variable** | **Section Completion Variable** | **Missing, comp=0** | **Missing, comp=1** | **Missing, comp=2** | **Missing, comp=3** | **% of Total Missingness from Non-completion** |
| --- | --- | --- | --- | --- | --- | --- |
| age | cs_s1 | n/a | 0/21 | 0/561 | n/a | n/a |
| race_whit | cs_s1 | n/a | 1/21 | 15/561 | n/a | n/a (no comp=0 group) |
| gad_q1 (outcome) | cs_s2 | 23/23 | 12/25 | 2/534 | n/a | 62.2% |
| gad_q2 (outcome) | cs_s2 | 23/23 | 12/25 | 6/534 | n/a | 56.1% |
| chronic_12 | cs_s2 | 0/23 | 0/25 | 0/534 | n/a | n/a |
| social_1 (primary predictor -- Twitter) | cs_s6 | 125/125 | 1/10 | 1/408 | 0/39 | 98.4% |
| social_2 (Instagram) | cs_s6 | 125/125 | 1/10 | 2/408 | 0/39 | 97.7% |
| social_3 (Facebook) | cs_s6 | 125/125 | 1/10 | 4/408 | 0/39 | 96.2% |
| social_4 (other social media) | cs_s6 | 125/125 | 1/10 | 7/408 | 0/39 | 94.0% |
| social_5 (other internet/video) | cs_s6 | 125/125 | 4/10 | 15/408 | 0/39 | 86.8% |
| politics | cs_s6 | 125/125 | 5/10 | 2/408 | 39/39 | 73.1% |
| income | cs_s6 | 125/125 | 5/10 | 25/408 | 39/39 | 64.4% |

*Note. age and chronic_12 have zero missing values. race_whit's missingness (16 respondents) occurs entirely among people who at least partially or fully completed Section 1, indicating genuine item nonresponse (e.g., "prefer not to answer") rather than attrition. The five social media battery items share an identical set of 125 respondents missing due to non-completion of Section 6, but are not perfectly identical overall: item-level nonresponse among the 408 Section 6 completers rises across the battery, from 1 (social_1, Twitter) to 2, 4, 7, and 15 (social_5, other internet/video), suggesting mild item-specific reluctance or fatigue toward the end of a repetitive question battery, layered on top of the dominant attrition mechanism. politics was missing for all 39 respondents in the comp=3 category, a pattern not observed for any social_* item or income, suggesting a distinct routing effect specific to that item rather than general end-of-survey attrition. gad_q1/gad_q2 show a similar attrition-dominant pattern to the social media items, though with somewhat more item-level nonresponse among Section 2 completers (2 and 6 of 534, respectively).*

# **Table S3.** Little's MCAR Test Results

Little's (1988) MCAR test was conducted separately for (1) the primary exposure and core covariates, and (2) the outcome (GAD-2) items, given differing missing-data patterns across these two variable sets (see Methods). Extended missing value codes were collapsed to standard missing prior to testing.

| **Variable Set** | **N** | **Chi-square** | **df** | **p-value** |
| --- | --- | --- | --- | --- |
| Exposure + core covariates (social_1, age, race_whit) | 582 | 22.5 | 5 | .<.001 |
| Outcome, GAD-2 items (gad_q1, gad_q2, age, race_whit) | 582 | 47.5 | 12 | <.001 |

*Note. Both tests reject the null hypothesis of missing completely at random (MCAR) at p < .05. Considered alongside the attrition decomposition in Supplementary Table 2, this pattern is consistent with data missing at random (MAR) conditional on survey completion status, rather than MCAR or missing not at random (MNAR).*

**References**

Little RJ. A test of missing completely at random for multivariate data with missing values. J Am Stat Assoc. 1988;83(404):1198-1202.
